# Disabling an oncogenic transcription factor by targeting of control kinases

**DOI:** 10.18632/oncotarget.25971

**Published:** 2018-08-17

**Authors:** Christopher R. Vakoc, Alex Kentsis

**Affiliations:** Molecular Pharmacology Program, Sloan Kettering Institute, New York, NY, USA; Department of Pediatrics, Memorial Sloan Kettering Cancer Center, New York, NY, USA; Cold Spring Harbor Laboratory, Cold Spring Harbor, NY, USA

**Keywords:** leukemia, kinase, therapy, transcription factor

Dynamic and developmental regulation of gene expression represents an essential feature of all living cells. Kinase-mediated phosphorylation or nuclear localization of transcription factors is a well-recognized means of control of gene expression in mammals. For example, this process underlies the cellular effects of growth factors and their induction of expression of immediate/early genes as part of the “serum response” [1]. Likewise, original studies of cis-regulatory elements controlling gene expression revealed distinct kinase signaling pathways controlling the assembly of enhanceosomes [2].

In cancer, dysregulation of gene expression is responsible for most of the observed cellular phenotypes, including unrestrained proliferation, resistance to cell death, metastasis, and many others [3]. This occurs as a consequence of somatic mutations of genes encoding kinases and other signaling enzymatic mediators, factors regulating chromatin state and accessibility, and transcription factors themselves. Indeed, most of the recurrent pathogenic mutations affecting oncogenes and tumor suppressor genes in blood cancers such as acute myeloid leukemia involve factors that directly regulate transcription and gene expression [4].

**Figure 1:**
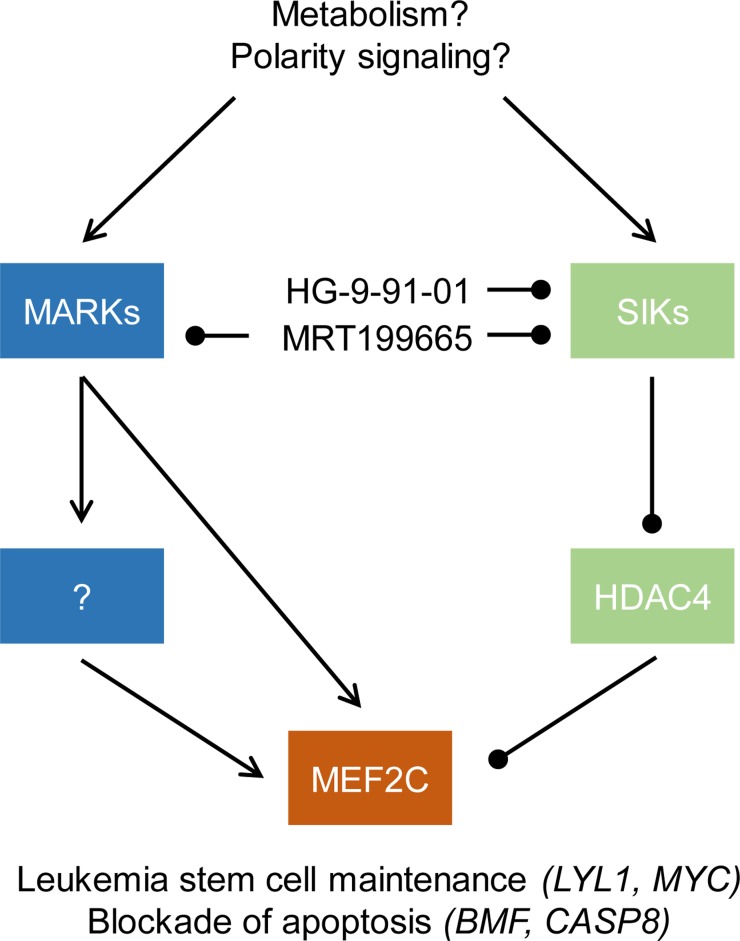
Kinase-dependent dysregulation of MEF2C in acute myeloid leukemia gene expression programs regulating leukemia stem cell maintenance, resistance to apoptosis and chemotherapy resistance MEF2C is controlled by the MARK and SIK kinases, which can be selectively inhibited using selective tool compound inhibitors HG-9-91-01 and MRT199665.

However, translation of these insights into improved therapies for patients has been hindered by the pharmacologic challenges of targeting oncogenic transcription factors or loss of tumor suppressors. Recently, we and others have employed high-resolution functional genomic and proteomic methods to define specific molecular dependencies and biochemical activities that distinguish leukemia cells from healthy blood progenitor cells [5, 6]. Building on prior work [7, 8], these studies convergently have led to the discovery of aberrant activation of the MADS box transcription factor MEF2C in a distinct subset of acute myeloid leukemias. Using functional CRISPR screening, Tarumoto et al discovered that specific AML cell lines exhibit a shared dependency on the aberrant transcriptional activity of MEF2C, and the SIK kinases that phosphorylate its negative regulator HDAC4 [5]. By using functional proteomics, Brown et al identified phosphorylation of MEF2C by the MARK kinases that cause its aberrant activation, leading to enhanced leukemia cell survival, stem cell maintenance, and resistance to apoptosis and chemotherapy [6]. Both of these mechanisms can be pharmacologically targeted using emerging selective inhibitors of the SIK and MARK kinases (Figure 1). The ability to identify leukemias with aberrant MEF2C activation and use selective kinase inhibitors to modulate its functions has immediate potential for improved diagnosis and therapy of this disease. For example, we foresee future clinical trials in which patients with MEF2C activation are prospectively treated with investigational targeted therapies to overcome chemotherapy resistance.

Intriguingly, though the functions of MARK and SIK kinases in hematopoiesis and leukemia pathogenesis are currently undefined, these kinases share common regulatory mechanisms and signaling pathways in other tissues, such as cell metabolic and polarity signaling. It is possible that SIK and MARK signaling pathways in AML comprise a coherent mechanism of aberrant activation of MEF2C. Alternatively, they may stem from distinct modes of leukemic activation, leading to convergent and/or redundant oncogenic functions. Nonetheless, SIK and MARK knockout mice are viable, and while they have endocrine and immune defects, SIK and MARK deficiency appears to be largely compatible with normal hematopoiesis and blood development [9, 10]. This suggests that their pharmacologic targeting may have a compelling therapeutic index, as supported by genetic CRISPR modeling of MEF2C phosphomimetic and non-phosphorylatable mutants in mice *in vivo* [6]. The discovery that genetic or pharmacologic blockade of SIK/MARK activity in leukemia cells largely phenocopies direct targeting of the MEF2C transcription factor raises an exciting possibility that kinase-mediated regulation of transcription factors have broader utility for cancer drug development.

In summary, these findings emphasize the importance of kinase-dependent dysregulation of transcription factor control in acute myeloid leukemia, substantiating its clinical investigation for improved diagnosis and therapy. This work also highlights the opportunities for biological discovery afforded by the use of improved functional genomic and proteomic methods. We anticipate that future integration of these technologies will yield important insights into the molecular mechanisms of cancer pathogenesis, including the definition of additional mechanisms of gene control and dysregulation in human cancer. In particular, it is possible that elucidation of additional enzymatic regulators of transcription factor functions by way of acetylation, methylation and other post-translational modifications will yield new pharmacologic strategies to target ‘undruggable’ targets such as MYC and numerous other oncogenic proteins in human cancer.
